# Comparison of the myometrial transcriptome from singleton and twin pregnancies by RNA-Seq

**DOI:** 10.1371/journal.pone.0227882

**Published:** 2020-01-17

**Authors:** Sarah Arrowsmith, Yongxiang Fang, Andrew Sharp

**Affiliations:** 1 Harris-Wellbeing Preterm Birth Research Centre, Institute of Translational Medicine, University of Liverpool, Liverpool, United Kingdom; 2 Centre for Genomic Research, University of Liverpool, Liverpool United Kingdom; 3 University of Liverpool and Liverpool Women’s NHS Foundation Trust, members of Liverpool Health Partners, Liverpool, United Kingdom; Brown University, UNITED STATES

## Abstract

Preterm birth is recognized as the primary cause of infant mortality worldwide. Twin pregnancies are significantly more at risk of preterm birth than singleton pregnancies. A greater understanding of why this is and better modes of treatment and prevention are needed. Key to this is determining the differing pathophysiological mechanisms of preterm birth in twins, including the role of the myometrium and premature uterine contraction.

We performed RNA sequencing (RNA-Seq) of human myometrium from singleton and twin pregnancies at term (> 37^+0^ weeks) and preterm (< 37^+0^ weeks), collected during pre-labour Caesarean Section. RNA-Seq libraries were prepared from polyA-selected RNA and sequenced on the Illumina HiSeq 4000 platform. Differentially expressed genes (DEGs), GO (Gene Ontology) and KEGG (Kyoto Encyclopedia of Genes and Genomes) pathway enrichment were conducted using R software. Significance was determined with a false discovery rate–adjusted *P* value of <0.05. Only 3 DEGs were identified between gestational age-matched singleton and twin myometrium and only 1 DEG identified between singleton term and twin preterm tissues. Comparison of singleton preterm myometrium with twin term myometrium however, revealed 75 down-regulated and 24 up-regulated genes in twin myometrium. This included genes associated with inflammation and immune response, T cell maturation and differentiation and steroid biosynthesis. GO and KEGG enrichment analyses for biologically relevant processes and functions also revealed several terms related to inflammation and immune response, as well as cytokine-cytokine receptor interaction and chemokine receptor signalling. Data indicate that little or no differences exist in the transcriptome of singleton and twin myometrium when matched for gestational age. The significant up- and down-regulation of genes identified between preterm singleton and twin myometrium at term may point to transcriptome changes associated with the chronic levels of uterine stretch in twin pregnancy or genes associated with the myometrium transitioning to labour onset.

## Introduction

Preterm birth is defined as delivery before 37 weeks gestation and occurs at a rate of around 11% of all births worldwide [1]. It is the single biggest cause of neonatal mortality and morbidity with almost half of preterm births being preceded by preterm labour (PTL). Twin pregnancies represent approximately 3% of all births yet, in contrast to singleton pregnancy, twin pregnancy is characterised by much higher rates of preterm birth: Nearly 60% of all twins are born before 37 weeks and around 20% deliver before 34 weeks[1, 2]. Twin pregnancies therefore contribute disproportionately to the rates of preterm birth and adverse neonatal and infant outcomes and place a greater public health, economic and emotional burden on society.

As with singleton pregnancies, the aetiology of preterm birth in twins is likely to be multifactorial but risk factors predisposing to preterm birth in twins may be different to that of a singleton pregnancy [3, 4]. Infection or short cervix are known risk factors for preterm birth [5] and are likely to be common to both pregnancies, whilst others such as uterine (over) distension and early activation of contractile pathways may be more significant in twins. Endocrine differences may also exist, resulting in part from double the quantity of fetal hormones from having more than one fetus, each producing hormones in the fetal hypothalamic-pituitary-adrenal-placental axis and in the case of dichorionic twins, the effects of increased overall placental mass [3].

Uterine over-distention or ‘stretch’ which refers to the mechanical stress on the uterine wall following the increase in uterine volume, has long been thought to contribute to preterm labour onset: In humans, placement and inflation of a balloon extra-amniotically can induce abortion in the second trimester and induce uterine contractions at term or postpartum [6–8]. Interestingly, women randomized to larger volumes of balloon have significantly shorter time from induction to delivery [9]. Increased intrauterine volume using intraamniotic balloons in non-human primates also initiated preterm labour in 50% of animals [10]. Hence in twin pregnancy, or pregnancies complicated by polyhydramnios (excess amniotic fluid) or macrosomic (large) fetus, the resulting over-distention of the myometrium may contribute to the higher rates of PTL in these patients. Stretching of human myometrium *in vitro* is also linked to upregulation of contraction associated proteins such as cyclooxygenase 2 (COX-2) [11] and the oxytocin receptor (OTR) [12], pro-inflammatory factors such as IL-8 [13] as well as release of cytokines from myometrium [14] which are key inflammatory mediators involved in labour onset (preterm or term).

As is the case for singleton pregnancy, predicting and preventing spontaneous PTL and birth are important goals for optimising outcomes in twin pregnancies. Once labour has been initiated, there are a limited number of effective and safe treatments (tocolytics) to prevent birth [15]. Current treatments however, do not substantially improve neonatal and maternal outcomes [16]. Preterm labour prevention strategies such as progesterone for women with short cervix and history of PTL have shown some promise in singleton pregnancy [17]. However, all interventions appear to be less effective in twin pregnancy [18]. There is therefore a need for improvement in current tocolytic treatments or a targeted/personalised treatment approach for singleton and twin pregnancy groups. However, this requires a greater understanding of the different mechanisms (if any) which bring about PTL in twins and singletons.

In myometrial function studies, small but significant differences in contractility of twin myometrium have been observed including a heightened response to oxytocin [19] and differences in the response of twin pregnancy myometrium to some tocolytics and progesterone when contractions were stimulated with oxytocin [20, 21]. Together this suggests that there may be differences in the expression pattern of a number of proteins associated with contraction and its modulation in twins. Recently, TREK1 expression was implicated in maintaining uterine quiescence following prolonged stretch in twin pregnancy myometrium [22]. Studies involving gene expression in twin pregnancy myometrium however are few in number [10, 22, 23] and the mechanisms of PTL in twins is relatively understudied.

Previous studies examining the expression of mRNA or proteins in both humans and animals, show that there are marked changes in myometrial gene expression which precede the onset of labour [24]. A number of studies have sought to explore these changes at the whole genome level using DNA microarrays in both humans [24–30] and rodents [31–33], or more recently have adopted an RNA sequencing (RNA-Seq) approach [34–36]. RNA-Seq provides an open, unbiased method to explore transcriptomic changes at the genome level for a given cell or tissue such as in response to treatment or differences between patient groups. To date in human myometrium, it has been successfully applied to explore changes in the transcriptome in singleton pregnancies with labour onset [34, 36]. No study has yet compared the gene expression profiles of myometrium between singleton and twin pregnancies.

In this paper we present the results of the first RNA-Seq study comparing gene expression in myometrium from pre-labour twin and singleton pregnancies matched for preterm or term gestation. Our objective was to identify differences in the myometrial transcriptome between singleton and twin pregnancies which would point to a unique gene activation pattern (possibly attributed to increased uterine stretch) and provide insight into the early activation of contraction pathways that may underpin aberrant contractility in preterm labour in multiple pregnancy.

## Methods

### Myometrial sample collection

All samples were obtained with informed written consent and the study was approved by the local research ethics committee (North West 3 Research Ethics Committee- Liverpool East, Ref. 10/H1002/49) and had Institutional review board approval from the Research and Development department at Liverpool Women’s Hospital NHS Foundation Trust. Women were approached by a research midwife during Caesarean section (CS) booking appointments in pre-operative clinic or by a doctor during a routine scan appointment in the fetal medicine unit at Liverpool Women’s Hospital. Recruitment took place between February 2014 and October 2017.

Criteria for inclusion in the study was singleton or twin pregnancy booked for elective (pre-labour) CS delivery between 32 and 39 weeks of gestation where the estimated due date had been determined by ultrasound scan during pregnancy booking (10-12weeks). Women were excluded if they were found to have any medical condition e.g. diabetes, hypertension or preeclampsia or receiving any medication.

Samples were obtained by knife biopsy from the upper lip of the lower uterine segment during surgery, following delivery of the baby/ies and prior to oxytocin administration. Samples were immediately placed into Hanks balanced salt solution, transferred to the laboratory where they were inspected under a dissection microscope. Biopsies were cleared of small blood vessels, decidua, membrane and scar tissue (if present) to ensure a homogeneous cell type i.e. myometrium, was collected. Small pieces of myometrium were then placed into RNA later for 24 hours at 4°C and stored at -80°C prior to RNA extraction.

Samples were divided into 4 groups (n = 6 per group) according to gestation at delivery and multiplicity of pregnancy: Singleton Preterm (SP) and Twin Preterm (TP) delivering before 37+0 weeks gestation and Singleton Term (ST) and Twin Term (TT) delivering after 37+0 weeks gestation. Reasons for CS included: Previous CS or previous traumatic vaginal delivery (PTVD, n = 5), breech presentation (n = 4), maternal request (n = 7), poor fetal growth (n = 3), previous preterm delivery resulting in CS delivery (n = 2) or maternal reason unrelated to pregnancy (n = 3). A summary of maternal characteristics according to gestational age group are presented in Table 1.

**Table 1:** Summary characteristics of women with singleton and twin pregnancy by gestation group.

	Preterm			Term			All groups
	Singleton	Twin	*P* value	Singleton	Twin	*P* value	*P* value^#^
Mean gestation at delivery (days) (95% CIs)	251 (241–260)	249 (234–262)	0.75	268 (266–270)	266 (264–268)	0.70	**0.001**
Mean maternal age (years) (95% CIs)	33 (27.6–37.8)	33 (25.4–39.8)	0.98	30 (21.9–37.7)	34 (27.8–39.5)	0.30	0.712
Mean maternal height (cm) (SD)	1.70 (0.08)	1.66 (0.8)	0.47	1.64 (0.06)	1.66 (0.07)	0.98	0.443
Mean maternal weight (kg) (SD)	66.6 (10.4)	69.2 (6.6)	0.62	82.3 (23.2)	73.1 (13.2)	0.42	0.297
Mean BMI (kg/m_2_) (SD)	23.3 (4.9)	25.2 (4.0)	0.47	30.5 (7.7)	27.3 (4.7)	0.40	0.175
Mean birthweight (grams) (SD)	2726.7 (638.4)	4495.8 (646.4)	**0.001**	3354.0 (355.4)	5525.0 (426.1)	**<0.001**	**<0.001**
Median parity (IQR)	1 (0–1)	1 (0–2)	0.58	1 (0–3)	1 (1–3)	0.58	0.817
*Para 0 (n)*	1	3	0.050	1	0	0.549	0.196
*Para 1 (n)*	4	0		3	3		
*Para >1 (n)*	1	3		2	3		
Reason for CS							
*Previous CS (n)*			**0.040**	2		0.176	**0.014**
*PTVD (n)*				2	1		
*Breech (n)*		2			2		
*Maternal request (n)*		3		1	3		
*Medical condition unrelated to pregnancy (n)*	3						
*Poor fetal growth (n)*	1	1		1			
*Previous preterm delivery + CS (n)*	2						

Gestation at delivery, maternal age, BMI and birthweight data within each gestational age group (preterm or term) and between all 4 groups were compared by independent T-test and One-way ANOVA (^#^) respectively. Median parity was compared by Mann Whitney U test or Kruskal Wallis test (^#^). Parity by group and reason for CS was compared by Pearson’s Chi square. P<0.05 was taken as significant. N = 6 for all groups. PTVD: previous traumatic vaginal delivery. Medical condition unrelated to pregnancy included: previous abdominal surgery, problematic urinary retention and labral tear to hips.

SD; Standard deviation, IQR, interquartile range

For both preterm and term groups, there was no difference in mean/median values of maternal age, maternal BMI, parity or gestational age at delivery between singleton and twin pregnancy women, indicating that the groups were well-matched demographically. As expected however, neonatal birthweight (which was combined birthweight in the case of twins), was significantly greater in both twin pregnancy groups compared to their gestational age-matched singleton counterparts. Overall in comparing all four groups, neonatal birthweight in Twin Term > Twin Preterm > Singleton Term > Singleton Preterm. The indications for CS delivery were also significantly different between groups. Preterm groups tended to require CS delivery for issues related to the fetus or maternal medical conditions (not related to pregnancy), whilst term deliveries were for previous CS, PTVD, breech presentation or maternal request (Table 1).

### RNA extraction and quality assessment

Eighty to one hundred milligrams of myometrium was homogenised in 1 mL TRIzol using an IKA Ultra Turrax homogenizer (Cole-Palmer, UK) and extracted using TRIzol plus RNA purification kit (Invitrogen, UK) according to the manufacturer’s instructions. The RNA was eluted in 30μL of nuclease-free water and stored at 80°C. The quantity and purity of RNA was determined using a Qubit RNA BR Assay Kit and fluorometer (Invitrogen, UK) and NanoDrop^®^ ND-1000 spectrophotometer (Thermo Scientific, UK). A_260/_A_280_ and A_260_/A_230_ absorbance ratios were between 1.8–2.0 and 2.0–2.2 respectively. RNA was treated with TurboDNAse (Invitrogen, UK) to remove any residual genomic DNA. RNA integrity was inspected using an Agilent 2100 Bioanalyser (Agilent Technologies, CA, USA). RNA with an integrity number (RIN) of ≥7.0 was considered acceptable.

### RNA-Seq analysis

#### cDNA library preparation and sequencing

Total RNA was submitted to the Centre for Genomic Research Facility, University of Liverpool, for RNA-Seq library preparation and sequencing. One microgram of DNA-free total RNA was selected for poly-A using NEBNext Poly (A) mRNA magnetic isolation module (New England Biolabs UK Ltd, UK). RNA-Seq libraries were prepared from the enriched RNA using the NEBNext Ultra Directional RNA library Prep kit for Illumina. Libraries were purified using AMPure XP beads. Each library was quantified using Qubit and the size distribution assessed using the Agilent 2100 Bioanalyser.

Libraries were pooled and assessed by a Qubit dsDNA HS assay kit and qPCR using the Illumina Library Quantification kit from Kapa on a Roche Light Cycler LC48011. Concentrations of template DNA were adjusted to 3nM and denatured with 0.1N NaOH. Denatured cDNA was further diluted to a final concentration of 300pM. Clustering of the DNA templates was performed using HiSeq 3000/4000 paired end cluster Kit with a cBot cluster generation system (Illumina, San Diego, USA) according to the manufacturer’s instructions. Libraries were sequenced on an Illumina HiSeq 4000 using sequencing by synthesis chemistry to generate 2 x 150bp paired end reads.

#### RNA-seq data processing

Sequence libraries for each sample were processed (base called and de-multiplexed) using CASAVA (version 1.8.2, Illumina) to produce sequence data in fastq format. Raw data were filtered to remove Illumina adaptor sequences using Cutadapt version 1.2.1 [37] with option ‘–O 3’ applied to trim adapters from reads if they matched the adapter sequence for 3 bp or more at the 3′ end. Low quality bases and reads shorter then 20bp were also removed from the data set using Sickle version 1.200.

The trimmed read pairs were aligned using TopHat2 version 2.1.0 [38] against the *H*. *Sapiens* genome reference (Ensembl GRCH38, release 93). Default settings were applied, except for the option–g 1. Reads aligning to the reference genome were counted using HTSeq-count version 0.6.1p1 [39].

### Differential gene expression analysis

The count data were normalised across libraries to account for differences in library size among samples by using trimmed mean M (TMM) values in edgeR with default parameters. Gene-wise dispersion parameters were estimated and then used for logFC (log_2_ fold change) estimating and testing. Differential expression analysis was carried out using edgeR package [40] on all the non-zero count genes identified by TopHat. Differentially expressed genes (DEGs) were extracted by applying the threshold false discovery rate (FDR) of less than 0.05 to adjusted *P* values, which were generated using Benjamini and Hochberg approach [41].

We performed the following 4 contrasts:

1. Singleton Preterm vs. Twin Preterm (SP vs. TP)
2. Singleton Term vs Twin Term (ST vs. TT)
3. Singleton Preterm vs Twin Term (SP vs. TT)
4. Singleton Term vs Twin Preterm (ST vs. TP)

Contrasts 1 and 2 were performed to examine for differences between singleton and twin pregnancies when matched for gestational age group. Contrasts 3 and 4 were performed to further examine for the effect of stretch on gene expression, with particular emphasis on contrast 3 which was assumed to have the most marked difference in levels of myometrial stretch.

### Functional analysis: KEGG pathway and GO term-based analysis

Kyoto Encyclopaedia of Genes and Genomes (KEGG) pathway analysis and Gene Ontology term (GO term) annotation for biological processes, molecular function and cellular components were detected using “gage” and “GOFunction” in R package respectively. *P* values were adjusted to correct the effects of multiple tests using the Benjamini-Hochberg approach where applicable [41]. Significantly enriched KEGG pathways and GO terms were identified as those with an adjusted *P* value <0.05.

### Data deposition

All sequence data produced in this study have been submitted to ArrayExpress, European Bioinformatics Institute. Raw data files can be accessed under accession number E-MTAB-8233 (https://www.ebi.ac.uk/arrayexpress/experiments/E-MTAB-8233/).

## Results

### Overview of RNA-Seq data

In total, across the 24 sample cDNA libraries, 1786.7 million reads were sequenced, producing an average of 37.2 million paired-end reads per sample. For all samples, post adapter and quality trimming, the percentage of unpaired reads was less than 0.75%. Unpaired reads were generally shorter than paired reads indicating that they lost more base pairs due to poor quality. Therefore, paired reads post-trimming were better quality than the individual forward or reverse reads. With respect to alignment, most of the reads were successfully aligned (91–93%) using TopHat2 (Table 2). All aligned reads fall in the exon regions of 42,356 genes which represents 72.55% coverage of the current hg38 (version 93) reference transcriptome (58,395 genes in total).

**Table 2:** Number of sequence reads per sample and percentage alignment to Tophat2.

Sample	Sequence reads (millions)	Aligned reads (TopHat2; %)	Aligned paired reads (%)
Singleton Preterm 1	134.0	94.93	92.74
Singleton Preterm 2	51.1	94.77	92.52
Singleton Preterm 3	56.3	94.44	92.14
Singleton Preterm 4	56.0	94.23	91.85
Singleton Preterm 5	69.2	94.77	92.61
Singleton Preterm 6	125.4	94.75	92.38
Twin Preterm 1	110.5	94.55	92.28
Twin preterm 2	78.1	94.53	92.25
Twin Preterm 3	106.7	94.68	92.43
Twin Preterm 4	56.7	95.08	92.99
Twin Preterm 5	107.1	94.14	91.77
Twin Preterm 6	65.5	94.87	92.65
Singleton Term 1	60.4	94.68	92.45
Singleton Term 2	85.3	94.2	91.89
Singleton Term 3	61.8	95.13	92.98
Singleton Term 4	70.6	95.17	93.06
Singleton Term 5	59.5	94.68	92.32
Singleton Term 6	60.5	94.36	92.03
Twin Term 1	53.4	95.18	93.09
Twin Term 2	60.0	95.33	93.21
Twin Term 3	91.5	95.02	92.88
Twin Term 4	70.4	94.35	92.0
Twin Term 5	49.0	94.66	92.37
Twin Term 6	58.7	94.83	92.55

### Assessment of variation in count data

**Fig 1:**
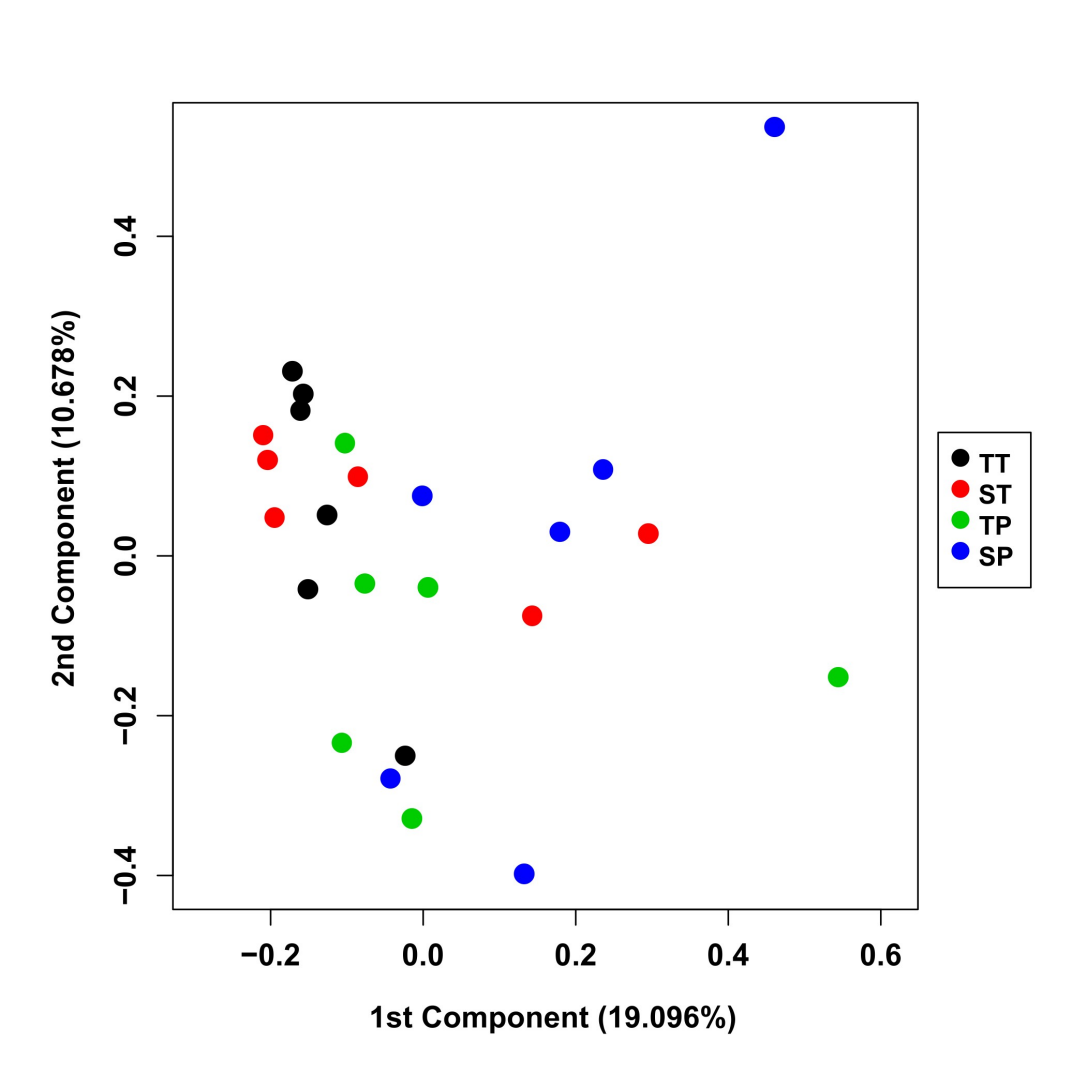
Principal component analysis (PCA) of the 1^st^ and 2^nd^ principal components from the 4 groups. Singleton Preterm (blue), Singleton Term (red), Twin Preterm (green), Twin Term (black). PCA was performed using the log10 count data from all libraries.

**Fig 2:**
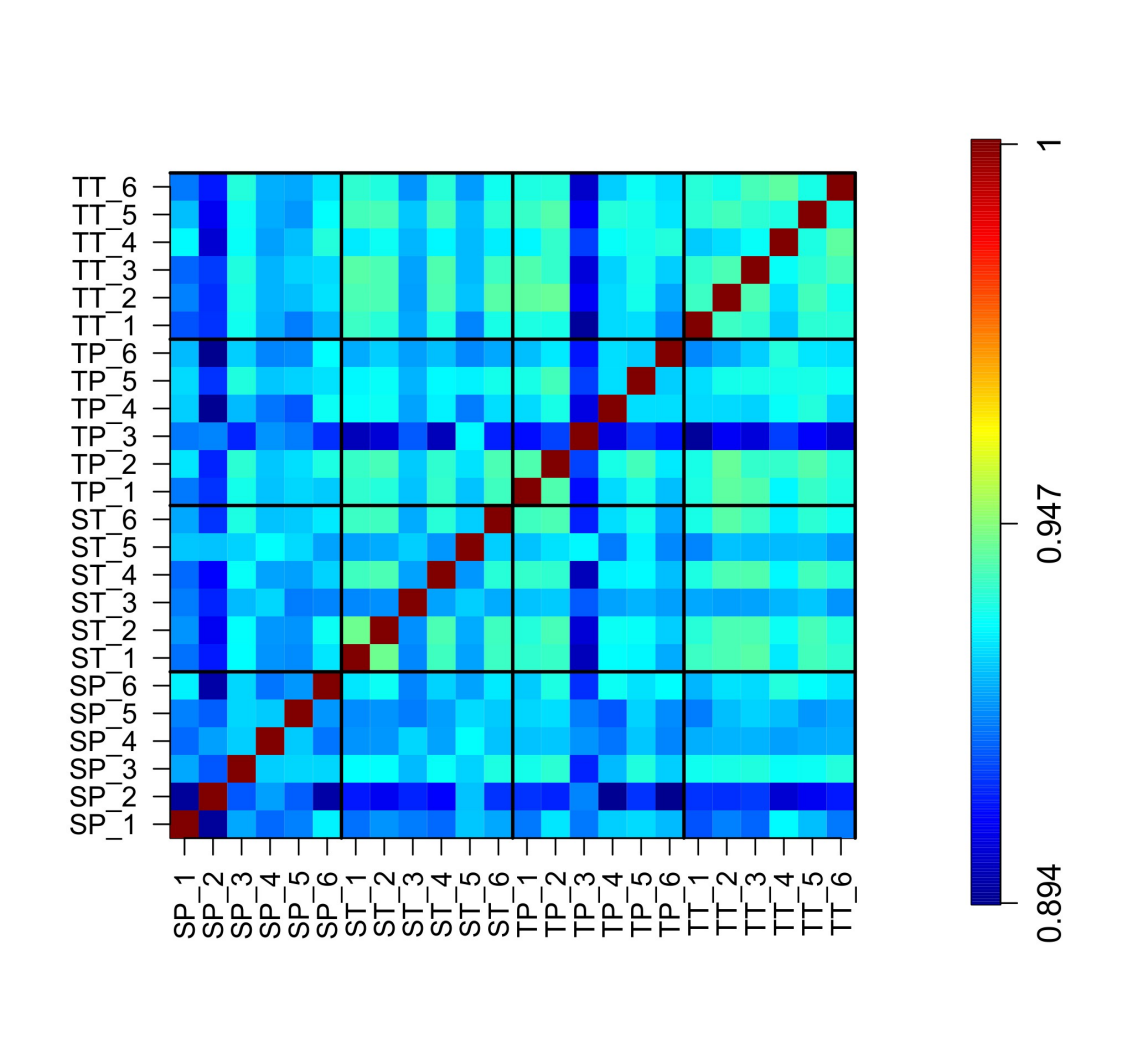
Sample correlation heat map of the correlation of all libraries. Each diagonal block (bottom left to top right) on the plot shows the correlation of samples within a sample group. Each of the remaining blocks shows the correlation between sample groups. SP; Singleton preterm, SP; Singleton term, TP; Twin preterm, TT; Twin term.

To assess clustering of groups, principal component analysis (PCA) of the first two principal components using the log10 count data from all libraries was also performed (Fig 1). This confirmed that sample groups did not cluster into distinct groups and could not be clearly separated. A sample correlation heat map of the correlation of all libraries was also created (Fig 2). This also showed that the correlations are similar both within and between groups. The lowest correlation amongst groups was >0.92. Together this indicted that the number of differentially expressed genes detected would be small.

### Identification of differentially expressed genes

When matched for gestational age (contrasts 1 and 2), we found only 3 genes which were differentially expressed between singleton and twin myometrium (Table 3). These genes correspond to: *ADRA1D*, alpha 1D adrenergic receptor, and *DPY19L2*, dpy-19-like 2 which were up-regulated in preterm twin pregnancy compared to preterm singleton pregnancy and *HLA-DQA2*, major histocompatibility complex class II, DQ Alpha 2, which was down-regulated in twin pregnancy at term compared to term singleton pregnancy.

**Table 3:** Numbers of DEGs identified in each contrast.

	Contrast			
	SP vs. TP	ST vs. TT	SP vs. TT	ST vs. TP
DEG total	2	1	99	1
DEG Up-regulated in twins	2	0	24	1
DEG Down-regulated in twins	0	1	75	0

SP: singleton preterm, TP: twin preterm, ST: singleton term, TT twin term

In both gestation groups (preterm and term), the combined birthweight of the neonates in twin pregnancies was significantly greater than the neonatal birthweight in singleton pregnancies (Table 1). The mean birthweight for the twin pregnancy group at term however, was more than double that of the mean birthweight of the singleton preterm group (5525g and 2726g respectively, Table 1). The additional stress placed on the myometrium in twin pregnancy resulting from the increased intrauterine volume has been suggested to contribute to higher incidence of preterm birth in this group. Using birthweight as an estimate of the level of uterine wall stress and hence a surrogate for investigating the effects of crude stretch on myometrial gene expression, we next compared the transcriptome data for twin pregnancies at term (TT) with singleton preterm pregnancies (SP) where the differences in birthweight and hence volume would be at its greatest.

**Fig 3:**
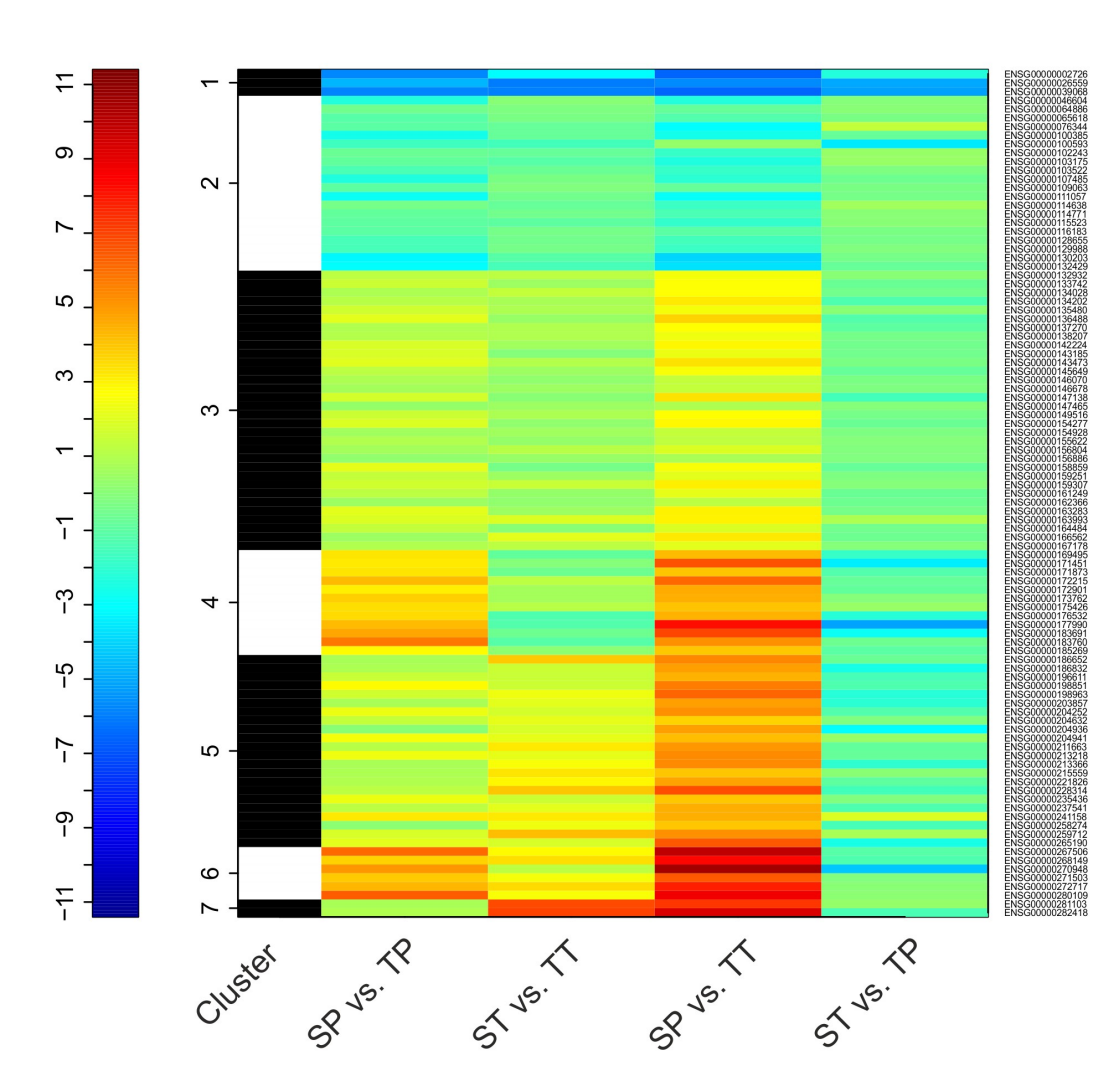
Heat map of log fold count (LogFC) of DE genes in the 4 contrasts. Detected DE genes were clustered based on the logFC value from all contrasts using the k-means method. SP; Singleton preterm, SP; Singleton term, TP; Twin preterm, TT; Twin term.

In total, the expression of 99 transcribed elements, including protein-coding transcripts, pseudogenes and long intergenic noncoding RNAs (lincRNAs) were significantly different in twin myometrium compared with singleton myometrium. Of these, 24 were at higher levels in the twin myometrium and 75 were at lower levels in the twin myometrium. Of the 24 transcripts expressed at a higher level in twin myometrium, 14 were known protein-coding genes. The remaining 10 genes contained 4 pseudogenes, 3 lincRNAs and 3 antisense genes. Of the 75 downregulated genes in twins, 70 were known protein coding genes. The remaining 5 gene biotypes included 4 antisense genes and one IG gene. Complete lists of up- and down-regulated protein-coding genes with a *P* value <0.05 and a Log_2_ fold change ≥1 are presented in Tables 4 and 5 respectively. The entire list of significantly DEGs is presented in S1 Table. Cluster analysis of the DEGs for all contrasts is shown in Fig 3.

**Table 4:** Protein coding transcripts up-regulated in term twin myometrium versus preterm singleton myometrium.

Gene symbol	Gene Name	LogFC	*P* value	FDR-adjusted *P* value
*KRT16*	Keratin 16	7.10	1.65E-06	0.0089
*MYH3*	Myosin Heavy Chain 3	3.34	8.69E-05	0.0434
*PLK5*	Polo Like Kinase 5	3.29	1.15E-04	0.0491
*ACP7*	Acid Phosphatase 7	3.11	2.61E-05	0.0280
*PDE11A*	Phosphodiesterase 11A	2.82	1.21E-05	0.0201
*EPHB1*	Ephrin type-B receptor 1	2.60	4.59E-05	0.0335
*KCNG1*	Potassium voltage-gated channel subfamily G member 1	2.43	2.55E-05	0.0280
*ADRA1D*	Alpha-1D-adrenergic receptor	2.26	1.88E-05	0.0265
*GSTM3*	Glutathione S-Transferase Mu 3,	2.01	1.13E-04	0.0491
*ACTC1*	Actin alpha cardiac muscle 1	2.00	1.24E-05	0.0201
*GSTM2*	Glutathione S-Transferase Mu 2	1.84	5.79E-05	0.0371
*WFDC1*	WAP Four-Disulfide Core Domain Protein 1	1.25	1.23E-05	0.0201

**Table 5:** Protein coding transcripts down-regulated in term twin myometrium versus singleton preterm myometrium.

Gene	Gene Name	LogFC	*P* value	FDR-adjusted *P* value
*NOTUM*	Palmitoleoyl-protein carboxylesterase	-11.40	3.39E-05	0.0296
*AOC1*	Amiloride-sensitive amine oxidase [copper-containing]	-10.77	1.02E-05	0.0194
*PSG3*	Pregnancy Specific Beta-1-Glycoprotein 3	-9.48	2.37E-06	0.0091
*XAGE2*	X Antigen Family Member 2	-8.99	2.82E-05	0.0280
*HSD3B1*	Hydroxy-Delta-5-Steroid Dehydrogenase, 3 Beta- And Steroid Delta-Isomerase 1	-8.80	2.73E-05	0.0280
*CSH2*	Chorionic Somatomammotropin Hormone 2	-8.39	3.26E-08	0.0005
*PRL*	Prolactin	-8.23	5.88E-05	0.0371
*LBP*	Lipopolysaccharide Binding Protein	-7.84	5.96E-05	0.0371
*CD177*	c-Kit	-7.41	2.09E-06	0.0089
*ALPP*	Alkaline Phosphatase	-7.23	1.69E-05	0.0265
*VGLL1*	Vestigial Like Family Member 1	-7.13	6.92E-05	0.0372
*PSG5*	Pregnancy Specific Beta-1-Glycoprotein 5	-6.95	3.41E-05	0.0296
*AADAC*	Arylacetamide Deacetylase	-6.70	3.47E-05	0.0296
*CSH1*	Chorionic Somatomammotropin Hormone 1	-6.61	3.50E-06	0.0123
*UPK1B*	Uroplakin 1B	-6.06	3.68E-05	0.0301
*CDH1*	Epithelial cadherin	-5.79	1.10E-04	0.0491
*STAR*	Steroidogenic Acute Regulatory Protein	-5.71	3.73E-05	0.0301
*MS4A3*	Membrane-spanning 4-domains subfamily A member 3	-5.70	3.96E-05	0.0305
*ADAMDEC1*	A Disintegrin And Metalloproteinase Domain-Like Protein Decysin-1	-5.65	1.14E-04	0.0491
*HLA-DQA2*	Major Histocompatibility Complex, Class II, DQ Alpha 2	-5.62	1.20E-10	0.0000
*KCNH1*	Potassium Voltage-Gated Channel Subfamily H Member 1	-5.48	7.26E-06	0.0166
*ATP8A2*	ATPase Phospholipid Transporting 8A2	-5.28	9.53E-05	0.0449
*CHI3L2*	Chitinase 3 Like 2	-5.28	1.80E-06	0.0089
*RBP4*	Retinol Binding Protein 4	-5.26	6.43E-05	0.0372
*PDZK1IP1*	PDZK1 Interacting Protein 1	-5.18	4.61E-05	0.0335
*GCM1*	Glial Cells Missing Homolog 1	-4.96	1.05E-05	0.0194
*PRR15*	Proline Rich 15	-4.89	2.18E-05	0.0277
*MMP1*	Matrix Metallopeptidase 1	-4.86	3.07E-05	0.0296
*LVRN*	Laeverin	-4.84	1.95E-07	0.0021
*COL17A1*	Collagen Type XVII Alpha 1 Chain	-4.80	2.42E-05	0.0277
*PRG2*	Proteoglycan 2	-4.75	5.51E-05	0.0364
*ISM2*	Isthmin 2	-4.55	2.25E-05	0.0277
*IL19*	Interleukin 19	-4.50	6.54E-06	0.0166
*NOG*	Noggin	-4.42	4.29E-05	0.0324
*ANXA8*	Annexin A8	-4.32	4.77E-05	0.0335
*DMKN*	Dermokine	-4.32	7.04E-05	0.0373
*PAPPA2*	Pappalysin 2	-4.28	8.23E-06	0.0166
*HTRA4*	HtrA Serine Peptidase 4	-4.26	2.09E-06	0.0089
*RORB*	RAR Related Orphan Receptor B	-4.24	5.40E-05	0.0364
*GNLY*	Granulysin	-3.97	2.34E-05	0.0277
*KRT7*	Keratin 7	-3.97	6.93E-05	0.0372
*ITGAD*	Integrin alpha-D	-3.62	2.84E-05	0.0280
*PLA2G7*	Phospholipase A2 Group VII	-3.61	3.27E-05	0.0296
*GATA3*	GATA Binding Protein 3	-3.41	1.01E-04	0.0472
*PCSK1*	Proprotein Convertase Subtilisin/Kexin Type 1	-3.36	2.84E-05	0.0280
*HLA-G*	Histocompatibility antigen, class I, G,	-3.24	1.13E-06	0.0080
*XCL2*	X-C Motif Chemokine Ligand 2	-3.17	3.32E-05	0.0296
*S100P*	S100 Calcium Binding Protein P	-3.11	1.07E-04	0.0489
*TNFRSF18*	Tumor necrosis factor receptor superfamily member 18	-2.99	1.14E-04	0.0491
*SCUBE1*	Signal Peptide, CUB Domain And EGF Like Domain Containing 1	-2.98	7.22E-05	0.0373
*APOE*	Apolipoprotein E	-2.93	3.84E-05	0.0301
*IL2RB*	Interleukin 2 Receptor Subunit Beta	-2.86	4.66E-06	0.0141
*IL21R*	Interleukin 21 Receptor	-2.85	6.71E-05	0.0372
*CXCR6*	C-X-C Motif Chemokine Receptor 6	-2.74	1.80E-05	0.0265
*GPR174*	G Protein-Coupled Receptor 174	-2.71	9.37E-05	0.0446
*KRT18*	Type I intermediate filament chain keratin 18	-2.68	2.98E-09	0.0001
*DSG2*	Desmoglein 2	-2.61	3.49E-05	0.0296
*POPDC3*	Popeye Domain Containing 3	-2.51	2.36E-05	0.0277
*GZMA*	Granzyme A	-2.45	8.85E-05	0.0434
*CD3E*	CD3-epsilon polypeptide	-2.37	5.47E-05	0.0364
*CD7*	Cluster of Differentiation 7	-2.31	9.06E-05	0.0436
*CCL5*	Chemokine (C-C motif) ligand 5	-2.06	2.24E-05	0.0277
*ISLR2*	Immunoglobulin Superfamily Containing Leucine Rich Repeat 2	-2.04	8.50E-05	0.0434
*TMEM200A*	Transmembrane Protein 200A	-1.52	3.76E-05	0.0301
*UCHL1*	Ubiquitin C-Terminal Hydrolase L1	-1.48	6.16E-05	0.0372
*FBXO32*	F-Box Protein 32	-1.45	5.58E-05	0.0364
*SEC11C*	Signal peptidase complex catalytic subunit	-1.37	7.45E-06	0.0166
*HLA-DOA*	HLA class II histocompatibility antigen, DO alpha chain	-1.34	4.67E-05	0.0335

The analysis identified differences in expression of a number of genes associated with inflammation and immune response, including: *HLA-DQA2*, *IL19*, *IL2RB*, *IL21R*, *CXCR6*, *CCL5*, *XCL2 and LBP* as well as *CD7*, *GATA3*, *TNFRS18*, *CDE3*, *GNLY and GZMA* which are genes involved in T cell maturation, differentiation and regulation of interactions with target cells. These were all identified as being down-regulated (log_2_ fold change ≤-1, p<0.05) in twin term myometrium. Other down-regulated genes included 3β-hydroxysteroid dehydrogenase (*HSD3B1)* and steroidogenic acute regulatory protein (*STAR)* which are involved in steroid hormone biosynthesis, including the catabolism of cholesterol to pregnenolone and the conversion of pregnenolone to progesterone. Genes associated with regulation of contraction such as *KCNG1* and *KCNH1* members of the voltage–sensitive potassium (K_v_) channel family, the interstitial cells of Cajal-like cell marker, c-kit (*CD177*) and phosphodiesterase 11A (*PDE11A*) which is involved in the downregulation of cAMP and cGMP signalling were also identified as being differentially expressed in twin term myometrium.

Comparing term singleton myometrium with preterm twin myometrium (contrast 4) however, only identified one gene which was differentially expressed. This corresponded to, carbonic anhydrase 1 (*CA1*) which was found to be up regulated in preterm twin myometrium compared to term singleton myometrium.

### GO functional enrichment and KEGG pathway analysis

To systematically determine functional analyses and canonical pathways that the DEGs might be involved in, we planned to perform GO-term and KEGG pathway enrichment on those genes identified as being significantly up- or down-regulated in twin myometrium. As the number of DEGs identified in contrasts 1, 2 and 4 was small, the DEG data generated was not suitable for use in pathway analysis. We therefore focussed on contrast 3: Singleton preterm versus twin term myometrium.

Genes were assessed for biological process, molecular function and cellular component GO-term enrichment and KEGG pathway enrichment using “GOFunction” and “gage” in R software. The genes identified were assigned to 24, 8 and 16 functional groups in the different GO categories respectively (Tables 6–8). The top GO-term biological processes identified as being altered in twin term myometrium compared to preterm singleton myometrium included ‘immune response,’ ‘regulation of immune response’ and ‘inflammatory response’ (Table 6). Whilst molecular function and cellular component ontologies were associated mostly with ‘receptor binding’ and the ‘plasma membrane’ (Tables 7 and 8).

**Table 6:** Significant Gene Ontology terms identified for biological processes in twin term myometrium compared to singleton preterm myometrium.

GO-term ID	GO-term name: *Biological Process*	Number of genes linked to this term	% of total genes in the list (3,146)	Adjusted *P* value
GO:0006955	Immune response	302	9.60	7.78E-16
GO:0050776	Regulation of immune response	114	3.62	1.41E-12
GO:0006954	Inflammatory response	362	11.51	2.01E-11
GO:0070098	Chemokine-mediated signalling pathway	67	2.13	8.91E-06
GO:0032496	Response to lipopolysaccharide	160	5.09	0.000212
GO:0030198	Extracellular matrix organization	192	6.10	0.000218
GO:0030593	Neutrophil chemotaxis	60	1.91	0.000241
GO:0032729	Positive regulation of interferon-gamma production	43	1.37	0.000460
GO:0006935	Chemotaxis	114	3.62	0.000515
GO:0007165	Signal transduction	1010	32.10	0.000847
GO:0007166	Cell surface receptor signalling pathway	254	8.07	0.001405
GO:0042102	Positive regulation of T cell proliferation	57	1.81	0.002377
GO:0006968	Cellular defence response	54	1.72	0.002597
GO:0060907	Positive regulation of macrophage cytokine production	7	0.22	0.003061
GO:0018916	Nitrobenzene metabolic process	4	0.13	0.003911
GO:0045165	Cell fate commitment	43	1.37	0.007575
GO:0007169	Transmembrane receptor protein tyrosine kinase signalling pathway	87	2.77	0.010208
GO:0034097	Response to cytokine	47	1.49	0.010334
GO:0050702	Interleukin-1 beta secretion	8	0.25	0.017803
GO:0050679	Positive regulation of epithelial cell proliferation	56	1.78	0.017839
GO:0007157	Heterophillic cell-cell adhesion via plasma membrane cell adhesion molecules	44	1.40	0.022039
GO:0045060	Negative thymic T cell selection	11	0.35	0.026560
GO:2000503	Positive regulation of natural killer cell chemotaxis	6	0.19	0.044966
GO:0042110	T cell activation	44	1.40	0.045567

*P* value represents the Benjamini-Hochberg corrected *P* value

**Table 7:** Significant Gene Ontology terms identified for molecular function in twin term myometrium compared to singleton preterm myometrium.

GO-term ID	GO-term name: *Molecular Function*	Number of genes linked to this term	% of total genes in the list (1,083)	Adjusted *P* value
GO:0005102	receptor binding	331	30.56	7.51E-08
GO:0008201	heparin binding	153	14.13	0.00093
GO:0050839	cell adhesion molecule binding	61	5.63	0.00123
GO:0004872	receptor activity	194	17.91	0.01743
GO:0008009	chemokine activity	45	4.16	0.02070
GO:0004252	serine-type endopeptidase activity	178	16.44	0.02644
GO:0005539	glycosaminoglycan binding	18	1.66	0.02703
GO:0005178	integrin binding	103	9.51	0.02811

*P* value represents the Benjamini-Hochberg corrected *P* value

**Table 8:** Significant Gene Ontology terms identified for cellular component in twin term myometrium compared to singleton preterm myometrium.

GO-term ID	GO-term name: *Cellular Component*	Number of genes linked to this term	% of total genes in the list (14,993)	Adjusted *P* value
GO:0005887	integral component of plasma membrane	1323	8.82	8.87E-23
GO:0005886	plasma membrane	3604	24.04	1.19E-21
GO:0009986	cell surface	488	3.25	1.17E-12
GO:0005615	extracellular space	1195	7.97	7.96E-09
GO:0009897	external side of plasma membrane	179	1.19	1.21E-07
GO:0005576	extracellular region	1288	8.59	5.99E-07
GO:0005796	Golgi lumen	84	0.56	2.34E-06
GO:0005578	proteinaceous extracellular matrix	238	1.59	6.27E-05
GO:0070062	extracellular exosome	2690	17.94	0.00013
GO:0016021	integral component of membrane	3226	21.52	0.00047
GO:0097486	multivesicular body lumen	7	0.05	0.00057
GO:0042105	alpha-beta T cell receptor complex	5	0.03	0.00570
GO:0043202	lysosomal lumen	83	0.55	0.00716
GO:0005622	intracellular	510	3.40	0.01005
GO:0031093	platelet alpha granule lumen	55	0.37	0.03387
GO:0042101	T cell receptor complex	18	0.12	0.03477

*P* value represents the Benjamini-Hochberg corrected *P* value

Active KEGG pathways also included cytokine-cytokine receptor interaction, chemokine signalling, toll like receptor signalling and nod-like receptor signalling which are all important inflammatory pathways mapped to the ‘immune system’ and ‘signalling molecules and interaction’ biological categories (Table 9).

**Table 9:** Significant KEGG pathways identified in twin term myometrium compared to singleton preterm myometrium.

KEGG ID	Pathway name	Biological category	Log FC	*P* Value	FDR- adjusted P value
04060	Cytokine-cytokine receptor interaction	Signaling molecules and interaction	-7.536	1.35E-13	2.86E-11
04142	Lysosome	Transport and catabolism	-6.369	4.79E-10	3.38E-08
04612	Antigen processing and presentation	Immune system	-6.117	5.20E-09	2.20E-07
04650	Natural killer cell mediated cytotoxicity	Immune system	-5.284	1.44E-07	4.36E-06
04380	Osteoclast differentiation	Development	-5.059	4.08E-07	9.61E-06
04672	Intestinal immune network for IgA production	Immune system	-5.308	4.79E-07	1.01E-05
04514	Cell adhesion molecules (CAMs)	Signaling molecules and interaction	-4.774	1.63E-06	2.88E-05
04062	Chemokine signaling pathway	Immune system	-4.211	1.62E-05	0.00023
04610	Complement and coagulation cascades	Immune system	-4.285	1.77E-05	0.00023
04630	Jak-STAT signaling pathway	Signal transduction	-4.149	2.25E-05	0.00028
04660	T cell receptor signaling pathway	Immune system	-4.047	3.66E-05	0.00041
04620	Toll-like receptor signaling pathway	Immune system	-4.044	3.83E-05	0.00041
04640	Hematopoietic cell lineage	Immune system	-4.053	4.11E-05	0.00041
04145	Phagosome	Transport and catabolism	-3.579	0.00020	0.00181
04621	NOD-like receptor signaling pathway	Immune system	-3.016	0.00159	0.01297
00510	N-Glycan biosynthesis	Glycan biosynthesis and metabolism	-2.887	0.00244	0.01846
04144	Endocytosis	Transport and catabolism	-2.637	0.00435	0.03071
04340	Hedgehog signaling pathway	Signal transduction	-2.579	0.00570	0.03665
04670	Leukocyte transendothelial migration	Immune system	-2.520	0.00623	0.03884
00600	Sphingolipid metabolism	Lipid metabolism	-2.549	0.00644	0.03903

## Discussion

In this study, we examined for the first time, the human myometrial transcriptome from singleton and twin pregnancies at two gestational time points (preterm and term) using high throughput RNA sequencing. When matched for gestational age, we find only a few genes which are differentially expressed between groups which implies that there is little or no difference in the myometrial transcriptome of singleton and twin pregnancies, at least when length of pregnancy is controlled for. Our unbiased statistical analysis of the sequenced transcriptomes of the 24 analysed samples in the form of principal component analysis was not able to clearly distinguish between the 4 clinical subgroups; singleton preterm, twin preterm, singleton term and twin term, further reflecting that there is little difference between the transcriptomes of these samples.

Previous studies employing RNA-Seq in human myometrium, have successfully examined changes in genes associated with labour onset in singleton myometrium in which there is a clear difference in the transcriptome once labour has been initiated [34, 36]. The DEGs identified in these RNA-Seq studies also broadly reflect those of earlier microarray studies. RNA-Seq also has the advantage of increased sensitivity and is not limited to the complement of pre-selected probes present on an array. Hence, we are confident that a high throughput RNA sequencing approach was suitable for use in this study.

Previous data relating to changes in gene and protein expression in twin pregnancy has been restricted to studies of a single or few genes [10, 22, 23] particularly focussed around myometrial stretch. Whilst this is useful for pinpointing the role of specific genes and proteins in a particular pathway, it does not give an overall unbiased view or qualitative assessment of the global changes in mRNA expression within or between tissues at a given time, which we aimed to identify here.

During human pregnancy, the uterus grows and expands to accommodate the developing fetus. Early in gestation this is mainly via hyperplasia (increased number of uterine cells) and a small contribution from increased cell size (hypertrophy). Towards late pregnancy, growth of the fetus imposes tension on the uterine wall, inducing a number of biochemical and molecular changes in the myometrial cells [42, 43]. This stretch is thought to be the major stimulus for uterine growth [44] and is a continual process allowing for uterine remodelling. Stretch pressures placed on the uterine wall by the growing fetuses in animal models of pregnancy and labour has been shown to increase the expression of myometrial contraction-associated protein (CAP) genes including connexin 43 (gap junction protein) and the oxytocin receptor (OTR) as well as to changes in the number of extracellular matrix proteins and activation of focal adhesions [45]. Application of mechanical stretch to human myometrial cells *in vitro* is also associated with up-regulation of *OTR*, *COX2* and secretion of cytokines and *IL-8* [11–13], further supporting a role for myometrial stretch contributing to myometrial activation.

It is not unreasonable to expect changes in myometrial tension in twin pregnancies given they tend to have a 60% increase in overall fetal birthweight. The hypothesis surrounding myometrial stretch and PTL onset is that PTL may reflect an inability of the uterus to adapt to this continuous increase in volume. In multiple pregnancies therefore (and those complicated by polyhydramnios), whilst stretch is a normal process and an important stimulus for uterine growth, stretch may be excessive. Hence, twin pregnancies which are ‘stretched’ to a greater extent at an equivalent time point in gestation compared to singleton pregnancies, are associated with an increased risk of preterm labour and delivery [46]. Undoubtedly, duration of pregnancy is shorter with increasing numbers of fetuses [47]. A recent large Swedish retrospective cohort study also supports uterine distention playing a causal role in determining the timing of birth in uncomplicated spontaneous deliveries, as well as a role for increased uterine load, maternal height and uterine size [48]. In examining tissues which were matched for gestational age and for other characteristics (as reported here), one may therefore have expected to see changes in gene expression resulting from the assumed increase in pressure on the uterine wall in twin pregnancy.

Our data showing no change in term samples is in agreement with a study by Lyall *et al*., [23] who investigated the expression of key regulators of myometrial function including, connexin 43 and 26, prostanoid receptors, EP1-EP4 and Gαs, in term (~38 weeks) singleton and twin myometrium and found no difference in expression between the two groups. They concluded that the mechanisms whereby stretch can activate myometrial contraction are complex and hence other factors must be involved. Our gestational age-matched cohort RNA-Seq data however, did not reveal any other candidate genes that warrant further investigation: The *ADRA1D* gene encodes alpha-1D-adrenergic receptor which is a member of the G protein-coupled receptor superfamily. Adrenergic receptors activate mitogenic responses and regulate growth and proliferation of many cells hence its role in myometrial cell growth in twins could be supported. *HLA-DQA2* is involved in antigen processing and presentation of exogenous peptide antigens for CD4 T-cell recognition via MHC classs II molecules which are expressed in antigen presenting cells (B lymphocytes, dendritic cells, macrophages). Hence its expression here probably arises from other cells i.e. immune cells, present within the myometrial biopsy.

To further explore the potential effect of stretch on the myometrial transcriptome, we next examined for differences in gene expression between singleton preterm and twin term samples as in terms of birthweight, this reflects the greatest difference between groups (more than double) and hence the greatest assumed change in uterine wall stress and myometrial stretch. In doing so, we detected 99 DEGs of which 74 were known protein coding genes and included changes in pro-inflammatory genes, genes associated with regulation of myometrial contraction and genes involved in the de-novo synthesis of steroids such as pregnenolone and progesterone which are important for the maintenance of pregnancy. Interestingly these were all down-regulated in the twin group.

We acknowledge however, that the exact increases in uterine wall tension cannot be determined and note that whether the significant increase in intrauterine volume in twin pregnancies also affects uterine wall tension is questioned [49]. Is it therefore difficult to say whether these genes reflect the crude/long term effects of stretch on myometrial gene expression. The changes identified could also be due to changes in gestational age between the two groups. Whilst it was not the focus of our study, we addressed this by also performing a subset analysis to identify DEGs between preterm and term groups (data not shown). We found 32 DEGs between the singleton preterm and term groups, but, only 12 of these (~10%) overlapped with the DEGs from contrast 3. We found no changes between twin preterm and term samples. Hence, we do not think that differences in gestational age contribute extensively to the DEGs identified between preterm singleton and term twin myometrium.

Our analysis of Gene Ontology terms relating to biological processes and KEGG pathways between singleton preterm and twin term samples also revealed alterations in immune response, regulation of immune response and inflammation, as well as cytokine and chemokine signalling. The inflammatory genes identified however, were down-regulated in our twin group which is contradictory to stretch inducing pro-inflammatory gene expression as previously reported from *in vitro* studies examining the effects of stretch on human myometrial smooth muscle cells [10, 13, 14, 50]. One explanation for this could be that *in vivo*, the stretch applied is not acute and the myometrium may be responding to and compensating for the increasing stretch over time. The effects of chronic (long term) stretch such as anticipated in twins here is therefore likely to be different to the effects of the more acute mechanical stretch as used in studies investigating the effects of stretch *in vitro*.

Changes in protein function with increased stretch rather than levels of expression may also be playing a role and hence would not be detected in our study. For example, Aye *et al*, recently suggested that stretch of myometrium induces a change in the conformation of the transmembrane domains of the oxytocin receptor which facilitates signal transduction and leads to enhanced contractility even in the absence of oxytocin[51]. Hence in multiple pregnancy, stretch-induced activation of the OTR rather than increased OTR expression, may contribute to the higher rates of preterm birth in these women.

### Study limitations

One of the main limitations of this study is that samples were obtained from women undergoing ‘elective’ pre-labour CS. We chose to examine samples from these women with the intention of being able to study changes in gene expression between groups independently of changes that may occur with labour onset. We felt that using samples collected at the time of labour (preterm or term), irrespective of the initial mechanistic trigger, could be too biologically similar to distinguish changes in expression between sample groups. For example, the activation of similar final common pathways leading to labour such as inflammation, may mask any significant detectable changes between groups. To this end we also chose samples from women with similar base characteristics including gestational age (within gestation group), parity, height, weight and BMI and maternal age to further reduce non labour-associated variation between samples. In addition, therapeutically, any potential gene marker of PTL would need to be identifiable prior to the onset of labour to allow the greatest opportunity to intervene to prevent preterm delivery.

In choosing women pre-labour however, we were unable to fully match samples by indication for CS. This is because elective preterm deliveries are usually complex and performed to protect the health of the mother and/or fetus from continuing pregnancy, whilst elective CS at term are most likely due to fetal malposition, previous CS or complications in a prior delivery. In this study, we minimised the impact of indication for CS on study outcomes by only including preterm women with medical issues if their condition was unrelated to pregnancy, and excluded any woman on medication e.g. for diabetes or hypertension (all gestations).

We also hypothesised that this study would enable us to isolate changes in the transcriptome resulting from the extra biomechanical stress or ‘stretch’ placed on the myometrium, as anticipated in twin pregnancy. Whilst little or no changes were observed in our groups when matched for gestational age, our analysis of singleton preterm myometrium verses twin term myometrium (where the difference in neonatal birthweight is most significant) did detect some DEGs. However, we cannot rule out the effects of other factors e.g. hormonal differences (fetal or maternal) in multiple gestations being responsible for the differences observed. As our samples were from pre-labour women, we also do not know how ‘far off’ from labour the patients were and therefore cannot control for changes in gene expression which may be associated with the myometrium transitioning towards labour onset.

It is disappointing that the results of this study do not add much to our understanding of the different mechanistic triggers for preterm (and term) labour in twins verses singletons, should one exist. The high correlation between our samples (>0.92) and lack of sample group separation by principle component analysis supports that the real number of DEGs between groups is small. Increasing study sample size is therefore unlikely to increase the identification of DEGs between our gestational age-matched cohorts. Application of more stringent criteria for gene filtering (i.e. removing genes with zero counts in >25% of samples, did not significantly alter DEG number (data not shown) nor did it provide further sensible functional interpretation of our findings.

Examining expression from in-labour samples may yield greater differences. Hence, future studies should aim to examine the myometrial transcriptome changes associated with preterm labour onset in both singletons and twins, comparing samples from those in not in labour to those where spontaneous labour has begun. For example, Johnson’s group comparing twins in preterm labour versus no labour showed that protein levels of inflammatory cytokines and chemokines including TNFα, Il6, IL8 and CCL2 were increased in myometrial tissues [10] but they did not examine singleton tissues. By their nature, however, preterm in-labour tissues are more difficult to obtain since most women delivering preterm will do so vaginally. In addition, further examination of changes in gene expression according to parity (i.e. comparing nulliparous versus parous women) would also be informative, since parity is known to affect pregnancy and labour-specific processes.

In conclusion, twin pregnancy is associated with higher rates of preterm labour, possibly attributed to increased myometrial stretch and uterine over-distention. We find little or no difference in the transcriptome of singleton and twin pregnancy myometrium when matched for gestational age. In this regard we are therefore unable to conclusively add to previously suggested mechanisms, including uterine stretch, which are responsible for premature activation of myometrium in multiple pregnancy. Factors derived from other tissues such as the placenta, amnion and the fetus itself may be important, as well as examining samples from women undergoing spontaneous preterm labour.

## Supporting information

S1 Table Complete list of all DEGs identified (up- and down-regulated) in term twin myometrium versus preterm singleton myometrium (contrast 3), including all gene biotypes; protein-coding transcripts, pseudogenes, antisense genes and long intergenic noncoding RNAs (lincRNAs). (DOCX)
