# Supplementary material for: Comparison of the myometrial transcriptome from singleton and twin pregnancies by RNA-Seq
Source: PLoS One. 2020 Jan 17;15(1):e0227882. doi: 10.1371/journal.pone.0227882 (PMC6968856; doi:10.1371/journal.pone.0227882)
Supplement: S1 Table — (DOCX) [file pone.0227882.s001.docx]

S1 Table

| **gene_id** | **gene_symbol** | **gene_name** | **gene_biotype** | **log2FC** | **P Value** | **FDR** |
| --- | --- | --- | --- | --- | --- | --- |
| ENSG00000186832 | KRT16 | Keratin 16 | Protein coding | 7.096161214 | 1.65E-06 | 0.008855335 |
| ENSG00000228314 | CYP4F29P | Cytochrome P450 family 4 subfamily F member 29 pseudogene | Transcribed unprocessed pseudogene | 7.090123358 | 6.28E-05 | 0.037153691 |
| ENSG00000215559 | ANKRD20A11P | Ankyrin repeat domain 20 family member A11 pseudogene | Transcribed unprocessed pseudogene | 6.134851278 | 0.00011344 | 0.049096363 |
| ENSG00000270948 | MTDHP1 | Metadherin pseudogene 1 | Processed pseudogene | 4.308992569 | 4.82E-05 | 0.033471744 |
| ENSG00000282418 | AC092811.2 | Novel transcript | lincRNA | 4.012736935 | 1.87E-05 | 0.026511964 |
| ENSG00000109063 | MYH3 | Myosin Heavy Chain 3 | Protein coding | 3.335659308 | 8.69E-05 | 0.043416085 |
| ENSG00000185988 | PLK5 | Polo Like Kinase 5 | Protein coding | 3.287240381 | 0.000114754 | 0.049096363 |
| ENSG00000233304 | LINC01346 | Long intergenic non protein coding RNA 1346 | lincRNA | 3.112984265 | 0.000106964 | 0.048867997 |
| ENSG00000183760 | ACP7 | Acid Phosphatase 7 | Protein coding | 3.10531227 | 2.61E-05 | 0.0280017 |
| ENSG00000128655 | PDE11A | Phosphodiesterase 11A | Protein coding | 2.819282113 | 1.21E-05 | 0.0201261 |
| ENSG00000154928 | EPHB1 | Ephrin type-B receptor 1 | Protein coding | 2.596596458 | 4.59E-05 | 0.033471744 |
| ENSG00000026559 | KCNG1 | Potassium voltage-gated channel subfamily G member 1 | Protein coding | 2.430451605 | 2.55E-05 | 0.0280017 |
| ENSG00000171873 | ADRA1D | Alpha-1D-adrenergic receptor | Protein coding | 2.260761805 | 1.88E-05 | 0.026511964 |
| ENSG00000241158 | ADAMTS9-AS1 | ADAMTS9 antisense RNA 1 | antisense | 2.057432565 | 6.13E-06 | 0.016604163 |
| ENSG00000134202 | GSTM3 | Glutathione S-Transferase Mu 3, | Protein coding | 2.00808826 | 0.000113023 | 0.049096363 |
| ENSG00000159251 | ACTC1 | Actin alpha cardiac muscle 1 | Protein coding | 1.99691339 | 1.24E-05 | 0.0201261 |
| ENSG00000213366 | GSTM2 | Glutathione S-Transferase Mu 2 | Protein coding | 1.844866828 | 5.79E-05 | 0.037103459 |
| ENSG00000268149 | AC010336.4 | Novel transcript, antisense to MAP2K7 | antisense | 1.832293175 | 8.92E-05 | 0.043416085 |
| ENSG00000267506 | AC021683.2 | Novel transcript | lincRNA | 1.677244924 | 6.50E-05 | 0.037153691 |
| ENSG00000235436 | DPY19L2P4 | DPY19L2 pseudogene 4 | Transcribed unprocessed pseudogene | 1.510714116 | 7.23E-05 | 0.037325482 |
| ENSG00000103175 | WFDC1 | WAP Four-Disulfide Core Domain Protein 1 | Protein coding | 1.251914427 | 1.23E-05 | 0.0201261 |
| ENSG00000258274 | AC012085.2 | Novel transcript | antisense | 1.134681292 | 8.92E-05 | 0.043416085 |
| ENSG00000076344 | RGS11 | Regulator Of G Protein Signaling 11 | Protein coding | 0.843369158 | 6.15E-05 | 0.037153691 |
| ENSG00000177990 | DPY19L2 | dpy-19 like 2 | Protein coding | 0.7797424 | 7.98E-06 | 0.016604163 |
| ENSG00000171451 | DSEL | Dermatan Sulfate Epimerase Like | Protein coding | -0.778308439 | 7.62E-06 | 0.016604163 |
| ENSG00000158859 | ADAMTS4 | A disintegrin and metalloproteinase with thrombospondin motifs | Protein coding | -0.899854171 | 6.36E-05 | 0.037153691 |
| ENSG00000204252 | HLA-DOA | HLA class II histocompatibility antigen, DO alpha chain | Protein coding | -1.34194648 | 4.67E-05 | 0.033471744 |
| ENSG00000166562 | SEC11C | Signal peptidase complex catalytic subunit | Protein coding | -1.365194582 | 7.45E-06 | 0.016604163 |
| ENSG00000156804 | FBXO32 | F-Box Protein 32 | Protein coding | -1.446627193 | 5.58E-05 | 0.03635077 |
| ENSG00000154277 | UCHL1 | Ubiquitin C-Terminal Hydrolase L1 | Protein coding | -1.482638116 | 6.16E-05 | 0.037153691 |
| ENSG00000164484 | TMEM200A | Transmembrane Protein 200A | Protein coding | -1.515509344 | 3.76E-05 | 0.030074774 |
| ENSG00000167178 | ISLR2 | Immunoglobulin Superfamily Containing Leucine Rich Repeat 2 | Protein coding | -2.037050427 | 8.50E-05 | 0.043377001 |
| ENSG00000271503 | CCL5 | Chemokine (C-C motif) ligand 5 | Protein coding | -2.055097661 | 2.24E-05 | 0.027650795 |
| ENSG00000281103 | TRG-AS1 | Vestigial like Family member 1 | antisense | -2.238889863 | 2.00E-05 | 0.02739435 |
| ENSG00000173762 | CD7 | Cluster of Differentiation 7 | Protein coding | -2.30722882 | 9.06E-05 | 0.043595171 |
| ENSG00000198851 | CD3E | CD3-epsilon polypeptide | Protein coding | -2.371458933 | 5.47E-05 | 0.03635077 |
| ENSG00000145649 | GZMA | Granzyme A | Protein coding | -2.452811636 | 8.85E-05 | 0.043416085 |
| ENSG00000132429 | POPDC3 | Popeye Domain Containing 3 | Protein coding | -2.505781779 | 2.36E-05 | 0.027650795 |
| ENSG00000046604 | DSG2 | Desmoglein 2 | Protein coding | -2.614036248 | 3.49E-05 | 0.029576198 |
| ENSG00000111057 | KRT18 | Type I intermediate filament chain keratin 18 | Protein coding | -2.676242703 | 2.98E-09 | 6.30E-05 |
| ENSG00000147138 | GPR174 | G Protein-Coupled Receptor 174 | Protein coding | -2.711216782 | 9.37E-05 | 0.044612809 |
| ENSG00000172215 | CXCR6 | C-X-C Motif Chemokine Receptor 6 | Protein coding | -2.740932697 | 1.80E-05 | 0.026511964 |
| ENSG00000103522 | IL21R | Interleukin 21 Receptor | Protein coding | -2.848348719 | 6.71E-05 | 0.037153691 |
| ENSG00000100385 | IL2RB | Interleukin 2 Receptor Subunit Beta | Protein coding | -2.85984701 | 4.66E-06 | 0.014108355 |
| ENSG00000130203 | APOE | Apolipoprotein E | Protein coding | -2.926424965 | 3.84E-05 | 0.030087574 |
| ENSG00000159307 | SCUBE1 | Signal Peptide, CUB Domain And EGF Like Domain Containing 1 | Protein coding | -2.978395817 | 7.22E-05 | 0.037325482 |
| ENSG00000186891 | TNFRSF18 | Tumor necrosis factor receptor superfamily member 18 | Protein coding | -2.986719083 | 0.00011444 | 0.049096363 |
| ENSG00000163993 | S100P | S100 Calcium Binding Protein P | Protein coding | -3.113698198 | 0.000107298 | 0.048867997 |
| ENSG00000143185 | XCL2 | X-C Motif Chemokine Ligand 2 | Protein coding | -3.165721602 | 3.32E-05 | 0.029576198 |
| ENSG00000259712 | AC023906.5 | Novel transcript, antisense to MAPK6 | antisense | -3.222282116 | 6.42E-07 | 0.005438151 |
| ENSG00000204632 | HLA-G | Histocompatibility antigen, class I, G, | Protein coding | -3.240587645 | 1.13E-06 | 0.007986988 |
| ENSG00000175426 | PCSK1 | Proprotein Convertase Subtilisin/Kexin Type 1 | Protein coding | -3.358718703 | 2.84E-05 | 0.0280017 |
| ENSG00000107485 | GATA3 | GATA Binding Protein 3 | Protein coding | -3.406734586 | 0.000101399 | 0.047196083 |
| ENSG00000272717 | AC112236.2 | Novel transcript, antisense to SETD7 | antisense | -3.428048334 | 6.62E-05 | 0.037153691 |
| ENSG00000146070 | PLA2G7 | Phospholipase A2 Group VII | Protein coding | -3.605641975 | 3.27E-05 | 0.029576198 |
| ENSG00000156886 | ITGAD | Integrin alpha-D | Protein coding | -3.618425175 | 2.84E-05 | 0.0280017 |
| ENSG00000135480 | KRT7 | Keratin 7 | Protein coding | -3.969045817 | 6.93E-05 | 0.037153691 |
| ENSG00000115523 | GNLY | Granulysin | Protein coding | -3.972862283 | 2.34E-05 | 0.027650795 |
| ENSG00000198963 | RORB | RAR Related Orphan Receptor B | Protein coding | -4.240124809 | 5.40E-05 | 0.03635077 |
| ENSG00000169495 | HTRA4 | HtrA Serine Peptidase 4 | Protein coding | -4.262693191 | 2.09E-06 | 0.008855335 |
| ENSG00000116183 | PAPPA2 | Pappalysin 2 | Protein coding | -4.282471571 | 8.23E-06 | 0.016604163 |
| ENSG00000161249 | DMKN | Dermokine | Protein coding | -4.316881017 | 7.04E-05 | 0.037255625 |
| ENSG00000265190 | ANXA8 | Annexin A8 | Protein coding | -4.320923463 | 4.77E-05 | 0.033471744 |
| ENSG00000183691 | NOG | Noggin | Protein coding | -4.420614727 | 4.29E-05 | 0.032449259 |
| ENSG00000142224 | IL19 | Interleukin 19 | Protein coding | -4.495120953 | 6.54E-06 | 0.016604163 |
| ENSG00000100593 | ISM2 | Isthmin 2 | Protein coding | -4.554655608 | 2.25E-05 | 0.027650795 |
| ENSG00000186652 | PRG2 | Proteoglycan 2 | Protein coding | -4.751802901 | 5.51E-05 | 0.03635077 |
| ENSG00000065618 | COL17A1 | Collagen Type XVII Alpha 1 Chain | Protein coding | -4.795128853 | 2.42E-05 | 0.027650795 |
| ENSG00000172901 | LVRN | Laeverin | Protein coding | -4.835005644 | 1.95E-07 | 0.002065997 |
| ENSG00000196611 | MMP1 | Matrix Metallopeptidase 1 | Protein coding | -4.858693918 | 3.07E-05 | 0.029568948 |
| ENSG00000176532 | PRR15 | Proline Rich 15 | Protein coding | -4.887175009 | 2.18E-05 | 0.027650795 |
| ENSG00000137270 | GCM1 | Glial Cells Missing Homolog 1 | Protein coding | -4.962187031 | 1.05E-05 | 0.01935118 |
| ENSG00000162366 | PDZK1IP1 | PDZK1 Interacting Protein 1 | Protein coding | -5.181426671 | 4.61E-05 | 0.033471744 |
| ENSG00000138207 | RBP4 | Retinol Binding Protein 4 | Protein coding | -5.259328119 | 6.43E-05 | 0.037153691 |
| ENSG00000064886 | CHI3L2 | Chitinase 3 Like 2 | Protein coding | -5.278179014 | 1.80E-06 | 0.008855335 |
| ENSG00000132932 | ATP8A2 | ATPase Phospholipid Transporting 8A2 | Protein coding | -5.283440224 | 9.53E-05 | 0.044872044 |
| ENSG00000143473 | KCNH1 | Potassium Voltage-Gated Channel Subfamily H Member 1 | Protein coding | -5.47624116 | 7.26E-06 | 0.016604163 |
| ENSG00000237541 | HLA-DQA2 | Major Histocompatibility Complex, Class II, DQ Alpha 2 | Protein coding | -5.619633337 | 1.20E-10 | 5.10E-06 |
| ENSG00000211663 | IGLV3-19 | Immunoglobulin Lambda Variable 3-19 | IG V gene | -5.640102923 | 6.86E-05 | 0.037153691 |
| ENSG00000134028 | ADAMDEC1 | A Disintegrin And Metalloproteinase Domain-Like Protein Decysin-1 | Protein coding | -5.654954645 | 0.000113867 | 0.049096363 |
| ENSG00000149516 | MS4A3 | Membrane-spanning 4-domains subfamily A member 3 | Protein coding | -5.697623257 | 3.96E-05 | 0.030499563 |
| ENSG00000147465 | STAR | Steroidogenic Acute Regulatory Protein | Protein coding | -5.707463955 | 3.73E-05 | 0.030074774 |
| ENSG00000039068 | CDH1 | Epithelial cadherin | Protein coding | -5.789712531 | 0.000109645 | 0.049096363 |
| ENSG00000114638 | UPK1B | Uroplakin 1B | Protein coding | -6.064493142 | 3.68E-05 | 0.030074774 |
| ENSG00000136488 | CSH1 | Chorionic Somatomammotropin Hormone 1 | Protein coding | -6.609700056 | 3.50E-06 | 0.01233832 |
| ENSG00000114771 | AADAC | Arylacetamide Deacetylase | Protein coding | -6.698967734 | 3.47E-05 | 0.029576198 |
| ENSG00000204941 | PSG5 | Pregnancy Specific Beta-1-Glycoprotein 5 | Protein coding | -6.952010482 | 3.41E-05 | 0.029576198 |
| ENSG00000280109 | PLAC4 | Placenta enriched 4 | antisense | -6.976088358 | 4.00E-06 | 0.013043551 |
| ENSG00000102243 | VGLL1 | Vestigial Like Family Member 1 | Protein coding | -7.126549268 | 6.92E-05 | 0.037153691 |
| ENSG00000163283 | ALPP | Alkaline Phosphatase | Protein coding | -7.227747711 | 1.69E-05 | 0.026511964 |
| ENSG00000204936 | CD177 | c-Kit | Protein coding | -7.410988491 | 2.09E-06 | 0.008855335 |
| ENSG00000129988 | LBP | Lipopolysaccharide Binding Protein | Protein coding | -7.84040561 | 5.96E-05 | 0.037103459 |
| ENSG00000172179 | PRL | Prolactin | Protein coding | -8.230767875 | 5.88E-05 | 0.037103459 |
| ENSG00000213218 | CSH2 | Chorionic Somatomammotropin Hormone 2 | Protein coding | -8.392686441 | 3.26E-08 | 0.000460885 |
| ENSG00000203857 | HSD3B1 | Hydroxy-Delta-5-Steroid Dehydrogenase, 3 Beta- And Steroid Delta-Isomerase 1 | Protein coding | -8.800604542 | 2.73E-05 | 0.0280017 |
| ENSG00000155622 | XAGE2 | X Antigen Family Member 2 | Protein coding | -8.993736617 | 2.82E-05 | 0.0280017 |
| ENSG00000221826 | PSG3 | Pregnancy Specific Beta-1-Glycoprotein 3 | Protein coding | -9.481951069 | 2.37E-06 | 0.00912656 |
| ENSG00000002726 | AOC1 | Amiloride-sensitive amine oxidase [copper-containing] | Protein coding | -10.76507049 | 1.02E-05 | 0.01935118 |
| ENSG00000185269 | NOTUM | Palmitoleoyl-protein carboxylesterase | Protein coding | -11.39650448 | 3.39E-05 | 0.029576198 |
